# Radiofrequency regulation of the sphenopalatine ganglion in managing herpes zoster ophthalmicus neuralgia: A case series

**DOI:** 10.1097/MD.0000000000037884

**Published:** 2024-04-19

**Authors:** Min Cui, Na Zhang, Dong Wang, Lei Han

**Affiliations:** a Department of Pain Medicine, Central Hospital Affiliated to Shandong First Medical University, Jinan, People’s Republic of China; b Department of Pain Medicine, Jinan Zhangqiu District Hospital of Traditional Chinese Medicine, Jinan, People’s Republic of China.

**Keywords:** herpes zoster neuralgia, ophthalmic branch, radiofrequency regulation, sphenopalatine ganglion, trigeminal nerve

## Abstract

**Introduction:**

Trigeminal herpes zoster, which comprises 10% to 20% of cases of herpes zoster, often leads to severe pain in the ophthalmic branches. Current treatments, including drug therapy and minimally invasive interventions, have limitations; accordingly, there is a need to explore alternative approaches. This study aimed to evaluate the efficacy and safety of computerized tomography (CT)-guided pulsed radiofrequency of the sphenopalatine ganglion in patients with intractable trigeminal herpetic pain.

**Patient concerns:**

Three patients with intractable trigeminal ophthalmic zoster neuralgia were studied. All patients complained of bursts of headache, which occurred at least 10 times a day, usually in the periorbital and frontal regions. Conventional treatments, including oral medications and radiofrequency therapy targeting the trigeminal-semilunar ganglion and supraorbital nerve, could not sufficiently provide relief.

**Diagnosis:**

Two patients were diagnosed with herpes zoster in the ocular branch of the trigeminal nerve with conjunctivitis, while one patient was diagnosed with postherpetic neuralgia in the ocular branch of the trigeminal nerve.

**Interventions:**

This study employed a novel approach that involved CT-guided radiofrequency regulation of the pterygopalatine fossa sphenopalatine ganglion.

**Outcomes:**

In all three patients, pain relief was achieved within 1 to 3 days after treatment. During the follow-up, one patient had pain recurrence; however, its severity was ≈ 40% lower than the pretreatment pain severity. The second patient had sustained and effective pain relief. However, the pain of the third patient worsened again after 2 months. The average follow-up duration was 3 months. None of the enrolled patients showed treatment-related adverse reactions or complications.

**Conclusion:**

Our findings indicated that CT-guided radiofrequency regulation of the pterygopalatine fossa sphenopalatine ganglion was a safe and effective intervention for pain in patients with trigeminal ophthalmic zoster neuralgia, suggesting that it may be a therapeutic option if other treatments fail.

## 1. Introduction

In recent years, the annual incidence of herpes zoster has increased. Reactivation and replication of the herpes virus within the semilunar ganglion or trigeminal nerve cause edema and necrosis of neurons in the infected ganglia and nerves, resulting in trigeminal nerve-associated shingles,^[1,2]^ which remains a challenging and refractory neuropathic pain without effective treatment interventions. Herpes zoster ophthalmicus (HZO) occurs when the trigeminal ocular branch accumulates following the reactivation and replication of the latent varicella zoster virus in the trigeminal ganglion. Studies have indicated that 20% of all patients with herpes zoster have occurrences in the trigeminal ganglion, with the incidence in the first trigeminal nerve (V1) being ≈ 20 times higher than that in the V2 and V3 branches.^[3]^ A study conducted in the United States found that the annual incidence of eye complications from V1 shingles could reach 4%, which significantly affected the patients’ quality of life.^[4]^

HZO can progress into severe postherpetic neuralgia, which substantially affects the patient’s quality of life, especially in individuals aged > 60 years. Despite the use of appropriate medications and aggressive nerve blocks, including epidural blocks, persistent refractory pain in patients with shingles remains a challenge for physicians. The head and face are high-risk or sensitive areas for herpes zoster complicated with postherpetic neuralgia; further, the intensity of pain in the head and face postherpetic neuralgia is generally more severe, significantly impacting daily activities such as eating, washing, and sleeping. Given the unique location of the affected area, achieving continuous nerve block,^[5]^ spinal cord electrical stimulation, and intrathecal morphine pumps is challenging, which increases the treatment difficulty. Currently, their effectiveness in rapid analgesia, shortening the disease course, and reducing the incidence of postherpetic neuralgia is unsatisfactory.

Nerve pulse radiofrequency, a neuroregulatory therapy technique, has been widely used for the treatment of post-herpetic neuralgia. However, numerous reports have indicated its low efficiency or poor efficacy in post-herpetic neuralgia treatment.^[6]^ Interventional therapy involving pulsed radiofrequency (PRF) percutaneous ovale puncture of the trigeminal nerve semilunar segment can cause severe symptoms and complications, including keratitis, corneal ulcers, poor treatment outcomes, prolonged treatment courses, and residual neuralgia.

Our objective was to develop an effective treatment for herpes zoster neuralgia affecting the ocular branch of the trigeminal nerve. The pterygopalatine fossa is interconnected with surrounding cavities and fossae through the following eight channels: the orbital cavity via the suborbital fissure forward and above; the pterygopalatine canal backward and below; the oral cavity through the pterygopalatine canal downward; the nasal cavity via the sphenopalatine foramen and mandibular fissure laterally; and the infratemporal fossa through pterygopalatine fossa. In recent years, the regulation of nerves in the pterygopalatine fossa, particularly targeting the sphenopalatine ganglion and the maxillary nerve for the treatment of various head and facial pain, has regained attention among clinicians.^[7]^ Some researchers have suggested the utility of the stellate ganglion, semilunar ganglion of the trigeminal nerve,^[8]^ and its surrounding branches as therapeutic targets.^[9]^ However, there have been no reports of combined sphenopalatine ganglion radiofrequency therapy for intractable trigeminal herpetic pain.

Most patients with HZO often experience spontaneous needling, knife-like cutting, and burning pain in the affected region. Some patients also present with severe headaches at the frontal apex of the affected region and burst-tearing pain in the orbital and retroorbital regions, which occur numerous times a day for ≈ 5 to 10 minutes each time. During these episodes, patients exhibit signs of distress, including tears, shouting, holding their heads, crying, sitting, and lying down. The clinical presentation of pain in these patients resembles that of cluster headaches. This article describes three cases demonstrating the potential of PRF of the sphenopalatine ganglion for zoster-related trigeminal neuralgia characterized by severe burst-tearing headaches in the orbital and retroorbital regions.

## 2. Case series

Between February and August 2023, 3 patients were referred to our pain management department for zoster-related trigeminal neuralgia with severe burst-tearing headache.

### 2.1. Case 1

#### 2.1.1. Medical history

The patient, a 73-year-old woman, was admitted to the hospital with “right cephalic and facial postherpetic pain for > 4 months.” Four months prior, the patient developed pain on the right side of her head and face, which was accompanied by a rash. She visited the dermatology department of a local hospital and was diagnosed with herpes zoster. The patient received standard antiviral treatment in the dermatology department; however, the pain persisted, which was characterized by needle-like pain in the herps area. This pain was accompanied by a burst of headache in the periorbital, frontal, and parietal areas, with attacks occurring 10 to 20 times a day, each lasting ≈ 5 to 10 minutes, and accompanied by eye tearing on the affected side. Oral pregabalin and analgesics proved ineffective during the attacks.

**Figure 1.**
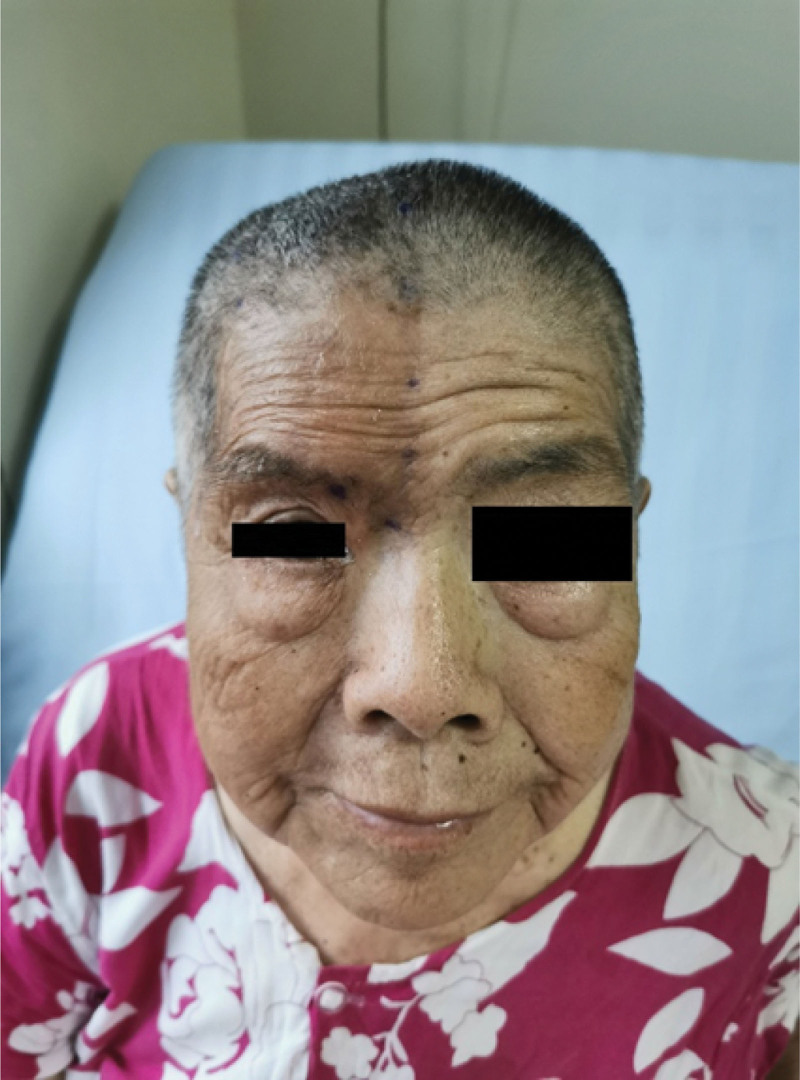
Herpes zoster affecting the right ophthalmic division of the right trigeminal nerve with pigmentation on the forehead and in the periorbital area.

The patient’s medical history included hypertension, coronary heart disease, atrial fibrillation, diabetes, osteoporosis, and hydronephrosis. She had previously undergone several surgeries, including left mastectomy, left knee joint replacement, and lumbar disc surgery. The patient denied any history of drug allergies. Her current home medication comprised 300 mg pregabalin and 40 mg dihydrocodeine. During the physical examination, postherpetic pigmentation was observed on the right side of the patient’s head, forehead, and upper eyelid without crossing the midline (Fig. 1). The skin prick sensation on the right side of the frontal surface was more sensitive than that on the left side, with touch-evoked pain and a numerical rating scale (NRS) score of 4 at rest and 8 to 9 during the outbreak. The admission diagnosis was postherpetic neuralgia in the ocular branch of the trigeminal nerve; accordingly, the patient was prescribed oral pregabalin 150 mg three times a day and a supraorbital nerve block under ultrasound guidance. This treatment provided slight relief from symptoms. Following consultation with the patient, we performed PRF of the sphenopalatine ganglion and supraorbital nerve under computerized tomography (CT) and ultrasound guidance, respectively. The plans, risks, benefits, and procedures of anesthesia were discussed, and informed consent was obtained.

#### 2.1.2. PRF procedure

**Figure 2.**
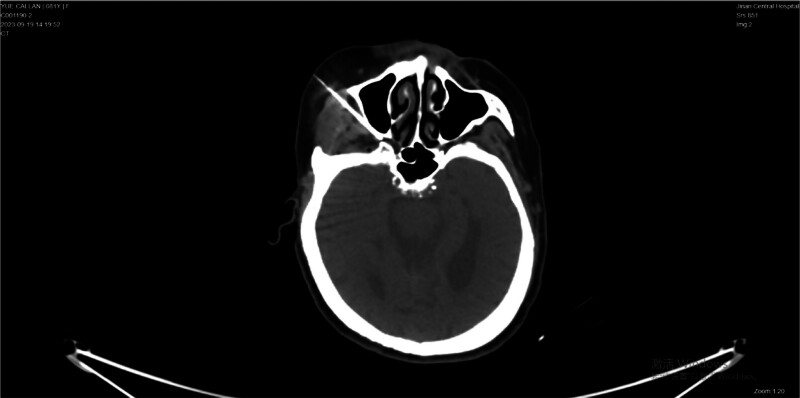
Representative CT image showing the electrode needle puncturing the pterygopalatine fossa. CT = computerized tomography.

The patient was placed supine on a CT scanner bed. CT was used to design and determine the puncture routes. Two milliliters of 1% lidocaine were used for skin localization; further, a 10-cm radiofrequency needle with a 5-mm active tip was inserted into the pterygopalatine fossa under CT guidance^[10]^ (Fig. 2). Sensory stimulation with 50-Hz current was applied to determine the correct position of the needle electrode. After confirming the needle position, three cycles of pulse radiofrequency at 42°C with a duration of 360 seconds (20 milliseconds current, 2 Hz, 45V) were performed. Subsequently, a mixture of 5 mL of 1% lidocaine with 0.5 mg mecobalamine and 7 mg betamethasone was injected into the pterygopalatine fossa.

#### 2.1.3. Results and follow-up

On postoperative day 1, the patient reported significant resolution of pain in the periorbital, frontal, and parietal areas. The outburst pain disappeared, and there was no lachrymation on the affected side; the NRS score was 2. The patient was followed up at weekly intervals for the first 3 postoperative months. Her pain returned at 1 postoperative month with an NRS score was 4, which was worse than that immediately after surgery. However, the pain degree was still relieved by ≈ 40% than that before the surgery.

### 2.2. Case 2

#### 2.2.1. Medical history

An 82-year-old woman presented with a complaint of “right frontal and orbital herps, with pain that persisted for more than one and a half months.” One and a half months prior, the patient visited the dermatology department of the hospital due to a rash on the right forehead and around the eye socket, which was accompanied by pain. The patient received antiviral drugs, and the rash became dry and crusty after 2 weeks; however, the pain did not significantly subside. The persistent pain included burning, needle-like sensations, and intermittent lightning-like pain, accompanied by a burst of headache in the periorbital, frontal, and parietal areas. The attacks occurred 20 to 30 times a day, with each episode lasting ≈ 3 to 5 minutes, accompanied by lacrimation in the right eye. Oral pregabalin and analgesics were ineffective during the attacks. The patient’s current home medication consisted of 300 mg pregabalin.

The patient had a medical history of hypertension and had previously undergone several surgeries, including lumbar disc surgery and right prosthetic femoral head replacement. Physical examination revealed postherpetic pigmentation on the right crown, forehead, and upper eyelid without crossing the midline; edema; droop on the right eyelid; laborious lifting; blurred vision; congestive conjunctival edema of the right eye; lacrimation; and skin acupuncture sensation on the right frontal surface, which was more sensitive than that on the left. The NRS score was 8. The admission diagnosis was herpes zoster in the ocular branch of the trigeminal nerve with conjunctivitis. Further, the admission treatment included prescribing oral pregabalin 150 mg twice daily and a supraorbital nerve block under ultrasound guidance, which provided slight relief from symptoms. Following consultation with the patient, a decision was made to perform PRF of the sphenopalatine ganglion and supraorbital nerve under CT and ultrasound guidance, respectively. The anesthesia plans, risks, benefits, and procedures were discussed, and informed consent was obtained.

#### 2.2.2. PRF procedure

The PRF procedure was the same as in Case 1.

#### 2.2.3. Results and follow-up

On postoperative day 3, the patient reported disappearance of the outburst pain, an 80% pain relief rate, and no lachrymation on the affected side. The redness and swelling of the conjunctiva were significantly relieved; further, the NRS score was 2. The patient was followed up at weekly intervals for the first 3 postoperative months. Her pain slightly increased, with the NRS score being 2 to 3.

### 2.3. Case 3

#### 2.3.1. Medical history

A 76-year-old man was admitted to the hospital with a complaint of “right-sided head and facial postherpetic pain persisting for 3 years.” Three years prior, the patient developed herpes accompanied by pain at the top of the right head and around the orbit, which yielded a diagnosis of herpes zoster. Antiviral treatment was administered, resulting in the resolution of the dry, scabbed rash. However, the patient continued to experience persistent pain at the right frontal apex and around the orbit, manifesting ≈ 10 times daily with light-like and burning sensations. Oral pregabalin and analgesics proved ineffective during these pain episodes. The patient underwent multiple supraorbital nerve blocks at a pain clinic and maintained a home medication regime of 300 mg pregabalin and 20 mg oxycontin.

The patient had a medical history of chronic obstructive pulmonary disease and had undergone colon cancer surgery in 2017. Upon physical examination, hyperalgesia was observed on the right frontal apex without crossing the midline, along with conjunctival hyperemia and a smaller cleft in the right eye. The NRS score was 5. The admission diagnosis was herpes zoster in the ocular branch of the trigeminal nerve with conjunctivitis. The treatment plan, which involved PRF of the sphenopalatine ganglion under CT guidance, was initiated upon consultation with the patient. The anesthesia plans, risks, benefits, and procedures were thoroughly discussed, and informed consent was obtained.

#### 2.3.2. PRF procedure

The PRF procedure mirrored that of Case 1.

#### 2.3.3. Result and follow-up

On postoperative day 3, the pain relief rate reached 70%, the conjunctival redness significantly subsided, and the NRS score was 3 (Table 1). Follow-up appointments were scheduled at weekly intervals for the initial 3 postoperative months. However, the patient reported the return of pain to the preoperative level after 2 months and declined a second PRF therapy due to personal reasons.

**Table 1:** Patient demographics, preoperative baseline data and primary outcomes.

Patient	Case 1	Case 2	Case 3
Age (yr)	73	82	76
Sex	Female	Female	Male
Days after zoster onset (mo)	4	1.5	36
Previous medications	Pregabalin 300 mg per day	Pregabalin 300 mg per day	Pregabalin 300 mg per day
	Dihydrocodeine 40 mg per day		Oxycontin 20 mg per day
Preoperative NRS	9	8	5
Postoperative NRS	2	2	2
Most recent NRS	4	3	4
Length of follow-up, mo	3	3	3
Complications	None	None	None

NRS = numerical rating scale.

## 3. Discussion

Acute herpes zoster trigeminal neuralgia and post-herpes zoster trigeminal neuralgia represent common forms of peripheral neuropathic pain, which are especially prevalent among older adults. Several studies have shown that prompt clinical interventions in patients with acute or subacute herpes zoster neuralgia can prevent and alleviate post-herpetic neuralgia.^[11]^ However, given its complex mechanism and challenging treatment, post-herpes neuralgia still lacks standardization. Currently, there have been scarce large-scale studies confirming the effectiveness of traditional surgical interventions, including semilunar ganglion radiofrequency, in treating herpes zoster neuralgia within the trigeminal nerve branch distribution area. Although current drug therapies have demonstrated certain efficacy, their effectiveness is suboptimal for patients with a protracted disease course, severe headaches, or recurrent pain episodes. The pathophysiology and clinical presentations of herpes zoster trigeminal neuralgia and primary trigeminal neuralgia significantly differ. Trigeminal postherpetic neuralgia is a form of refractory neuropathic pain that requires multifaceted approaches and combination therapies to improve pain, mood, and sleep disorders. Taken together, there is an urgent need for an effective treatment plan in clinical settings.

The exact mechanisms underlying post-HZO neuralgia remain unclear. The virus replicates in the ocular branch of the trigeminal nerve located in the cavernous sinus and extend to the orbit through the supraorbital fissure, where it can directly impair the optic nerve. The trigeminal somatosensory nerve fibers are located in the semilunar ganglion of the trigeminal nerve, with peripheral processes dividing into three branches: the optical, maxillary, and mandibular nerves. Therefore, clinical classification can be performed based on lesion sites and trigeminal nerve branches. HZO primarily occurs in the area of distribution of the ocular branch (V1) of the trigeminal nerve. Given that V1 nerves innervate numerous ocular and periocular structures, various ocular lesions and acute neuralgic episodes can occur. Typically, the skin around the eyes manifests erythema, blisters, and edema, which may extend to the forehead and scalp, forming ulcers and scars in severe cases. While HZO may lead to conjunctival edema, congestion, and sporadic bleeding, these symptoms often resolve within 1 week. Prodromal symptoms, including itching, numbness, and pain, may precede rashes. Depending on pain severity, recommended analgesic options include tricyclic antidepressants, gabapentin, pregabalin, topical lidocaine, or a combination of these drugs.^[12]^ Conventional tramadol and pregabalin may reduce pain for patients with V1 herpes zoster neuralgia who have severe lesions or pain; however, their efficacy is limited due to severe nerve injury. Therefore, it is important to establish methods for managing V1 herpes zoster neuralgia attacks and preventing progression to post-herpetic neuralgia.

Guidelines recommend a combination of multiple drugs for patients with post-HZO neuralgia given the ineffectiveness of single medication among these patients. Monitoring for adverse drug reactions, including gastrointestinal issues, cognitive impairment, gait abnormalities, and cardiotoxicity, is crucial during medication. Despite standardized drug treatment, some patients with postherpetic neuralgia of the trigeminal nerve remain unsatisfied with pain control or cannot tolerate adverse drug reactions, prompting the consideration of further interventional therapy combined with analgesia. Currently, this approach primarily involves trigeminal nerve block and regulatory therapy. The targets for nerve block treatment in post-HZO neuralgia usually include the stellate ganglion, semilunar ganglion of the trigeminal nerve, supraorbital nerve, and terminal nerve of the frontal face.^[9,13]^ Alternative nerve-blocking drugs comprise local anesthetics, glucocorticoids, nutraceutical drugs, ozone, and neurotropic drugs (such as doxorubicin). However, single nerve block therapy has short-lived efficacy, necessitating timely adjustments to individual treatment plans and often requiring the repetition of multiple or multi-site nerve block therapies combined with nerve regulation therapy.

PRF generates high voltage around the tissue through pulsed current, which regulates the plasticity of pain afferent pathways, reduces inflammatory mediators around damaged nerves, and activates the descending inhibitory pathway of the painful toe to produce analgesia. Strict control of the tip temperature of the exposed electrode (≤43°C) during PRF therapy is crucial to avoid damage to the motor nerves caused by the nerve thermal disassembly effect. PRF therapy boasts the advantages of being minimally invasive, safe, convenient, and repeatable, which have allowed it to be widely used for post-herpetic neuralgia treatment.^[14]^ Although numerous randomized controlled studies suggest that PRF, combined with other methods, can achieve improved efficacy for patients with post-herpetic neuralgia in common areas such as the chest and back with a disease course < 6 months, the efficacy may significantly diminish in the trigeminal nerve. For clinicians, the treatment of trigeminal herpes zoster neuralgia is relatively trickier. Some studies have found that the stellate ganglion, semilunar ganglion of the trigeminal nerve, or its surrounding branches are potential therapeutic targets^[15]^; however, the treatment effect is unsatisfactory. Accordingly, we aimed to find a better therapeutic target for relieving post-HZO neuralgia.

In our study, three patients with ocular branch herpes zoster experienced severe pain around the orbit and frontal region, accompanied by conjunctival edema, swelling, and non-spontaneous lacrimation. These symptoms closely resemble those associated with trigeminal autonomic headaches. Trigeminal autonomic cephalalgia is a common type of primary headache. Its typical onset involves the first trigeminal nerve and intracranial parasympathetic nerve on the same side; further, it includes conditions such as cluster headache, paroxysmal migraine, conjunctival injection, and lacrimation. Studies have shown that the ocular branch (V1 branch) of the trigeminal nerve receives pain stimulation from the forehead, eyes, dura, and larger cerebral vessels; subsequently, it transmits these signals to the receptive nucleus (trigeminal cervical complex) in the brainstem and upper cervical spinal cord. Ultimately, this information is conveyed to the pain neural matrix, which is a collection of brain regions through the thalamus that regulates various types of pain. The sphenopalatine ganglion, which is located in the pterygopalatine fossa, plays a crucial role in the trigeminal cerebrovascular system. Comprising parasympathetic and sympathetic nerve components, it is directly or indirectly connected to the facial body, visceral nerve fibers, superior salivary nucleus, and hypothalamus.^[16]^ Moreover, it has been implicated in the pathophysiology of cluster headaches.^[17,18]^ Ma et al^[10]^ reported the efficacy of CT-guided radiofrequency treatment of the sphenopalatine ganglion for trigeminal autonomic cephalalgia. Given the role of the pterygopalatine ganglion in the treatment of trigeminal autonomous headache, we decided to use it as a therapeutic target for post-HZO neuralgia. After consulting with the patient, we decided to perform PRF of the sphenopalatine ganglion under CT guidance. Following treatment, these patients reported improvements in their pain. The NRS scores after treatment ranged from 2 to 4. Two patients reported sustained improvement in pain with a mean follow-up of 3 months. No complications occurred.

### 3.1. Limitations

Our study has several limitations. First, we included a small sample size of only three patients. Second, this was not a randomized study. Accordingly, future large-scale prospective studies are warranted.

## 4. Conclusions

Our findings suggest that CT-guided radiofrequency therapy of the pterygopalatine fossa-sphenopalatine ganglion combined with radiofrequency regulation of the supraorbital nerve holds promise in achieving rapid relief for post-HZO neuralgia. While our findings demonstrate a favorable outcome within 3 months with minimal complications, it is essential to acknowledge the limitations of our small sample size. Accordingly, long-term follow-up studies and large-sample randomized controlled trials are warranted to comprehensively assess the safety and efficacy of this technique. This approach, if proven effective, could revolutionize the management of post-HZO neuralgia, offering a potential breakthrough for patients grappling with this challenging condition.

## Author contributions

**Conceptualization:** Na Zhang.

**Investigation:** Lei Han.

**Visualization:** Dong Wang.

**Writing – original draft:** Min Cui.

**Writing – review & editing:** Min Cui.

## References

[1] Bennett GJ Watson CP. Herpes zoster and postherpetic neuralgia: past, present and future. Pain Res Manag. 2009;14:275–82. 19714266 10.1155/2009/380384 PMC2734513

[2] Liesegang TJ. Herpes zoster ophthalmicus natural history, risk factors, clinical presentation, and morbidity. Ophthalmology. 2008;115(2 Suppl):S3–12. 18243930 10.1016/j.ophtha.2007.10.009

[3] Pelloni LS Pelloni R Borradori L. Herpes zoster of the trigeminal nerve with multi-dermatomal involvement: a case report of an unusual presentation. BMC Dermatol. 2020;20:12. 33126864 10.1186/s12895-020-00110-1 PMC7602315

[4] Pavan-Langston D Dohlman CH. Herpes zoster antivirals and pain management. Ophthalmology. 2008;115(2 Suppl):S13–20. 18243927 10.1016/j.ophtha.2007.10.012

[5] Choi EM Chung MH Jun JH. Efficacy of intermittent epidural dexamethasone bolus for zoster-associated pain beyond the acute phase. Int J Med Sci. 2020;17:1811–8. 32714084 10.7150/ijms.46038 PMC7378659

[6] Erdine S Ozyalcin NS Cimen A. Comparison of pulsed radiofrequency with conventional radiofrequency in the treatment of idiopathic trigeminal neuralgia. Eur J Pain. 2007;11:309–13. 16762570 10.1016/j.ejpain.2006.04.001

[7] William A Azad TD Brecher E. Trigeminal and sphenopalatine ganglion stimulation for intractable craniofacial pain – case series and literature review. Acta Neurochir. 2016;158:513–20. 26743912 10.1007/s00701-015-2695-y

[8] Wan CF Song T. Comparison of two different pulsed radiofrequency modes for prevention of postherpetic neuralgia in elderly patients with acute/subacute trigeminal herpes zoster. Neuromodulation. 2022;25:1364–71. 34008278 10.1111/ner.13457

[9] Javier J Wilton J Galluccio F. Pulsed radiofrequency for postherpetic trigeminal neuralgia: a case report. Cureus. 2022;14:e28913. 36237778 10.7759/cureus.28913 PMC9547084

[10] Ma Y Xu S Liu X. CT-guided thermocoagulation of the pterygopalatine ganglion for refractory trigeminal autonomic cephalalgia. Pain Ther. 2022;11:1071–7. 35749031 10.1007/s40122-022-00406-9 PMC9314506

[11] Fei Y Yao M Huang B. Efficacy of internal heat acupuncture combined with high-voltage long-duration pulsed radiofrequency on subacute postherpetic neuralgia: a retrospective study. Pain Res Manag. 2022;2022:1–7. 10.1155/2022/2180214 PMC9205724 35719198

[12] Pearl C Moxley B Perry A. Management of trigeminal neuralgia with botulinum toxin type A: report of two cases. Dent J (Basel). 2022;10:207. 36354652 10.3390/dj10110207 PMC9689410

[13] Xie K Liu S Huang B. Effects of supraorbital foramen variations on the treatment efficacy of radiofrequency therapy for V1 trigeminal neuralgia: a retrospective study. Pain Res Manag. 2020;2020:8142489. 32184911 10.1155/2020/8142489 PMC7061117

[14] Wang C Dou Z Yan M. Efficacy and safety of pulsed radiofrequency in herpes zoster related trigeminal neuralgia: a systematic review and meta-analysis. J Pain Res. 2023;16:341–55. 36756203 10.2147/JPR.S396209 PMC9901482

[15] Sun Z Liu L Liu H. Effect of CT-guided Gasserian ganglion block with local anesthetics and steroids on acute/subacute zoster-related trigeminal neuralgia: a multicenter retrospective study. J Pain Res. 2022;15:2303–13. 35974906 10.2147/JPR.S375257 PMC9375984

[16] Smith CR Helander E Chheda NN. Trigeminal nerve blockade in the pterygopalatine fossa for the management of postoperative pain in three adults undergoing tonsillectomy: a proof-of-concept report. Pain Med. 2020;21:2441–6. 32232479 10.1093/pm/pnaa062

[17] Assaf AT Hillerup S Rostgaard J. Technical and surgical aspects of the sphenopalatine ganglion (SPG) microstimulator insertion procedure. Int J Oral Maxillofac Surg. 2016;45:245–54. 26559753 10.1016/j.ijom.2015.09.023

[18] May A. Cluster headache: pathogenesis, diagnosis, and management. Lancet. 2005;366:843–55. 16139660 10.1016/S0140-6736(05)67217-0

